# Modelling changing population distributions: an example of the Kenyan Coast, 1979–2009

**DOI:** 10.1080/17538947.2016.1275829

**Published:** 2017-01-11

**Authors:** Catherine Linard, Caroline W. Kabaria, Marius Gilbert, Andrew J. Tatem, Andrea E. Gaughan, Forrest R. Stevens, Alessandro Sorichetta, Abdisalan M. Noor, Robert W. Snow

**Affiliations:** ^a^ Spatial Epidemiology Lab (SpELL), Université Libre de Bruxelles, Brussels, Belgium; ^b^ Department of Geography, Université de Namur, Namur, Belgium; ^c^ Spatial Health Metrics Group, KEMRI Wellcome Trust Research Programme, Nairobi, Kenya; ^d^ Fonds National de la Recherche Scientifique (F.R.S.-FNRS), Brussels, Belgium; ^e^ WorldPop, Department of Geography and Environment, University of Southampton, Southampton, UK; ^f^ Fogarty International Center, National Institutes of Health, Bethesda, MD, USA; ^g^ Flowminder Foundation, Stockholm, Sweden; ^h^ Department of Geography and Geosciences, University of Louisville, Louisville, KY, USA; ^i^ Nuffield Department of Clinical Medicine, Centre for Tropical Medicine and Global Health, University of Oxford, Oxford, UK

**Keywords:** Human population, distribution modelling, gridded population datasets, temporal change, Kenya

## Abstract

Large-scale gridded population datasets are usually produced for the year of input census data using a top-down approach and projected backward and forward in time using national growth rates. Such temporal projections do not include any subnational variation in population distribution trends and ignore changes in geographical covariates such as urban land cover changes. Improved predictions of population distribution changes over time require the use of a limited number of covariates that are time-invariant or temporally explicit. Here we make use of recently released multi-temporal high-resolution global settlement layers, historical census data and latest developments in population distribution modelling methods to reconstruct population distribution changes over 30 years across the Kenyan Coast. We explore the methodological challenges associated with the production of gridded population distribution time-series in data-scarce countries and show that trade-offs have to be found between spatial and temporal resolutions when selecting the best modelling approach. Strategies used to fill data gaps may vary according to the local context and the objective of the study. This work will hopefully serve as a benchmark for future developments of population distribution time-series that are increasingly required for population-at-risk estimations and spatial modelling in various fields.

## 1. Introduction

During the past decades, advances in GIS technologies have facilitated the mapping of the spatial distribution of human populations globally at an unprecedented level of detail (Dobson et al. 2000; Balk and Yetman 2004; Balk et al. 2006; Bhaduri et al. 2007; Linard and Tatem 2012; Doxsey-Whitfield et al. 2015). Existing large-scale gridded population datasets such as GRUMP, LandScan or WorldPop are typically based on a top-down approach, that is, an approach that starts with census data and disaggregates population counts within administrative units using a simple or more sophisticated dasymetric method (Dobson et al. 2000; Balk and Yetman 2004; Balk et al. 2006; Bhaduri et al. 2007; Linard and Tatem 2012; Doxsey-Whitfield et al. 2015). However, populations change rapidly and therefore make population datasets rapidly outdated. While spatial population predictions have been produced for the past or the near future, such predictions are generally produced by simply applying national population growth rates (Hay et al. 2004) or urban/rural population growth rates (Gaughan et al. 2013; Noor et al. 2014; Stevens et al. 2015) to current gridded population databases, and therefore do not include any subnational variation in historical population distribution trends. In addition, using urban/rural growth rates is heavily dependent on the urban definition used and ignores urban land cover (LC) changes over time. The gridded population of the World v4 database is the only one using two different rounds of censuses (circa 2000 and circa 2010) and therefore providing subnational variations in population distribution for the recent past (Doxsey-Whitfield et al. 2015).

Historical censuses or surveys often do not exist, are of poor quality or only available as very coarse aggregated numbers, especially in low income countries. One of the rare previous studies that attempted to map long-term population distribution changes is the History Database of the Global Environment (HYDE), whose main objective was to model long-term past land use dynamics. The HYDE project provides maps of population distribution for the period 10,000 BC to 2000 AD at a spatial resolution of 5 minutes (approximately 10 km at the equator) (Goldewijk, Beusen, and Janssen 2010; Goldewijk et al. 2011). Input population data include national historical population estimates from the literature, subnational population numbers from Populstat (Lahmeyer 2004) and national urban/rural fractions for the period 1950–2000 from the United Nations (United Nations Population Division 2014). The construction of the HYDE database inevitably relies on assumptions and extrapolations that induce significant uncertainties in the output, especially in data-scarce countries. The HYDE population density layers have been used to derive time-series of malaria incidence rates across large areas over the twentieth century (Snow et al. 2012; Tatem et al. 2013). However, while the accuracy of the HYDE database is acceptable for analyses at broad spatial and temporal scales, much more spatially and temporally detailed time-series of population distribution are needed in finer-scale studies.

In addition to historical aggregated population counts, historical ancillary data should ideally be used to increase the spatial resolution of historical population maps. Gaughan et al. (2016) recently produced temporally comparable, high-resolution datasets of gridded population distribution for mainland China using only time-invariant and temporally explicit covariates to model population distribution. The study demonstrated the importance of including urban development dynamics in the production of temporally comparable population datasets. Among the temporally explicit covariates used in this recent study, lights at night appeared as one of the most important predictor of population distribution (Gaughan et al. 2016). Such datasets have been shown to be good predictors of human presence at large-spatial scales (Sutton et al. 2001; Elvidge et al. 2007) and have the advantage of being multi-temporal, though limited to the last 2.5 decades (the first images were acquired in 1992). In addition, advances in methods allowing the automatic extraction of settlement extents from high-resolution remote sensing data recently led to the production of a global time-series of human settlement layers derived from Landsat data. The Global Human Settlement Layer (GHSL), developed and maintained by the Joint Research Centre of the European Commission, provides human settlement layers for the years 1975, 1990, 2000 and 2014, which open new promising opportunities in the mapping of human population distribution over longer time periods (Pesaresi et al. 2013, 2016; Freire et al. 2016).

Here we make use of these newly available settlement datasets, historical census data and recently developed population distribution modelling methods to reconstruct population distribution changes over 30 years across the Kenyan Coast, a data-scarce area characterized by high population growth rates. We also explore the methodological challenges and opportunities associated with the production of gridded population distribution time-series.

## 2. Materials and methods

### 2.1. Previously used methods

Methods used in the present paper are mainly based on previous work from Stevens et al. (2015) and Gaughan et al. (2016). Random forest (RF) models were estimated in order to provide dasymetric weighting layers to redistribute population counts within administrative units. An RF model is an ensemble, nonparametric modelling approach that combines individual regression trees and improves upon bagging (Breiman 2001; Liaw and Wiener 2002). The log-transformed population density of administrative units was used as a ‘response’ variable and values for covariates were obtained by aggregating values by administrative unit (using zonal means for each continuous dataset and class majorities for binary datasets). The resulting RF model is used to predict a pixel-level map of population densities, which is then used as a weighting layer for a standard dasymetric mapping approach, as described for the WorldPop datasets (Linard et al. 2012; Gaughan et al. 2013; Stevens et al. 2015). To produce gridded multi-temporal population datasets and analyse population changes, the RF modelling approach was applied to different census years (1990, 2000 and 2010) in mainland China (Gaughan et al. 2016). To make the population maps temporally comparable, covariates can only include time-invariant (e.g. elevation, slope, rivers) or temporally explicit (e.g. satellite-derived urban extents, lights at night) data (Gaughan et al. 2016).

### 2.2. Study area

**Figure 1.**
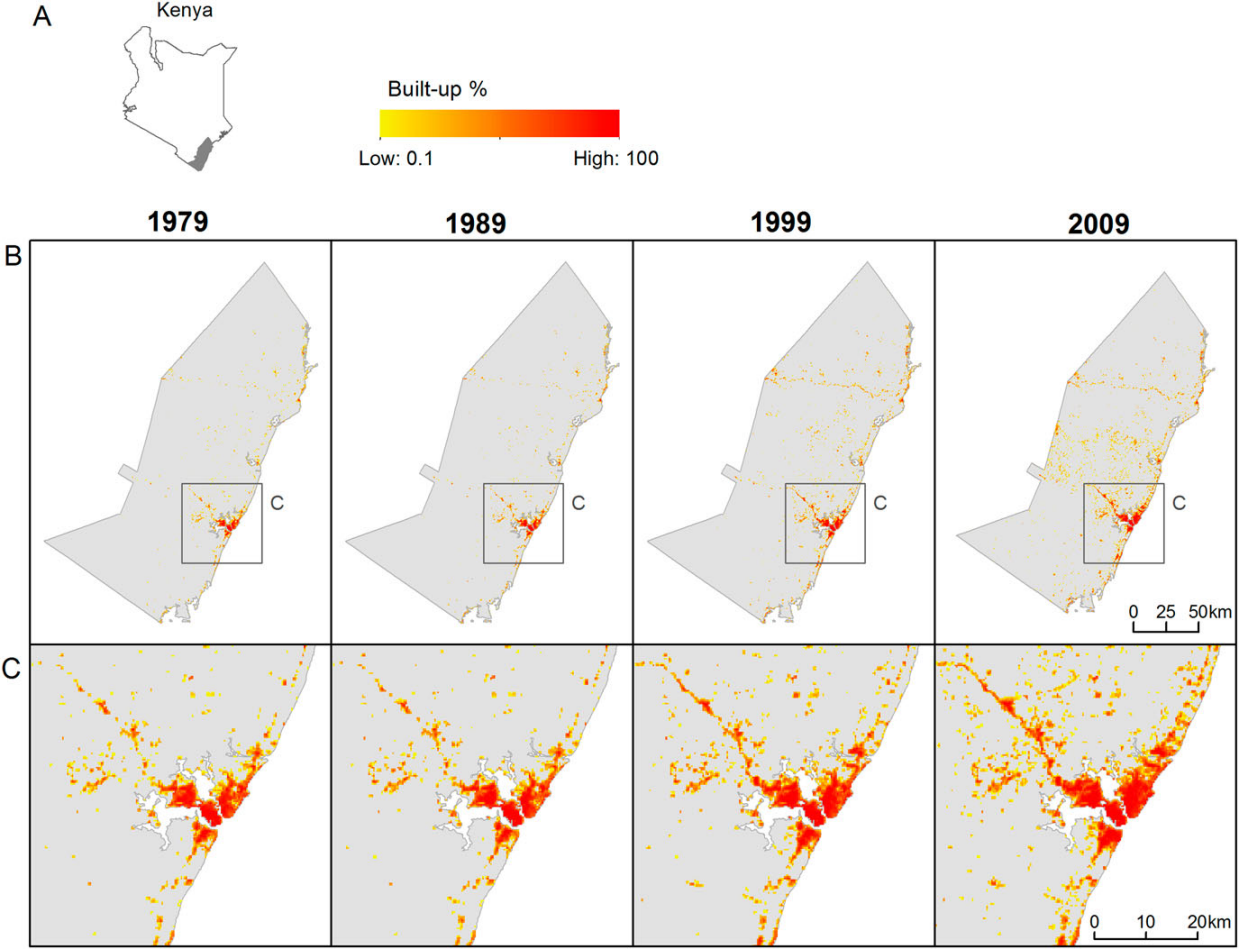
(a) Location of the study area in Kenya. (b and c) Estimated percentage of built-up area for the four census years (1979, 1989, 1999 and 2009) in logarithmic scale for (b) the whole study area and (c) close-ups around Mombasa (grid cell resolution is 3 arc seconds, or ∼100 m at the equator).

The study area covers the coastal districts of Kenya (Kilifi, Malindi, Mombasa and Kwale, according to the 1999 district boundaries) and currently represents about 7% of the Kenyan population (Figure 1(a)). The study area includes the second largest urban agglomeration of Kenya, Mombasa, which reached 1 million inhabitants in 2012 (United Nations Population Division 2014). Administrative divisions in Kenya are classified into six different levels: administrative unit level 0 (ADM-0; national), ADM-1 (province), ADM-2 (district), ADM-3 (division), ADM-4 (location), ADM-5 (sub-location) and ADM-6 (enumeration areas).

### 2.3. Census population data

The first complete Kenyan censuses were undertaken in 1948 (Colony & Protectorate of Kenya 1953), 1962 (MFEP 1964) and 1969 (MFEP 1970) and then were repeated every 10 years (CBS 1979, 2001; MEPD 1981; KNBS 2010). During the 1969 census, the smallest administrative units used were sub-locations (ADM-5), and from 1979, these were further divided into enumeration areas (ADM-6) for the purpose of census logistics. An ADM-6 unit represents approximately 100 households or 500 persons (MEPD 1981).

Unfortunately, ADM-5 data for the 1969 census, or ADM-6 maps and data for the 1979 and 1989 censuses cannot be located within the Kenya National Bureau of Statistics (KNBS). However, ADM-5 boundaries can be mapped and linked to data for all censuses since 1979, although these boundaries have changed between censuses. It was possible to reconcile ADM-5 boundaries (*n* = 279) between the 1999 and 2009 censuses. ADM-5 data for the 1989 census were available from the World Resources Institute (*n* = 212). Boundaries were cleaned to ensure that coastlines, district boundaries and other obvious matches corresponded with the boundaries of the 1999/2009 census data. The 1979 data were not available in any GIS format; however, reports from KNBS provide tabulated information at ADM-5 level (MEPD 1981). Here we used matching names of sub-locations (ADM-5) and locations (ADM-4) to configure the best possible match to the 1989 census ADM-5 boundaries resulting in 191 polygons that could be linked to de facto population counts. Data at the finest resolution (ADM-6) are only available for the 1999 census. Table 1 summarizes the finest census data available for each census year since 1979.

**Table 1.** Summary of finest census population data available for each census year and total de facto population for the study area (Kenyan coastal districts), 1979–2009.

Census year	Finest administrative unit level	Administrative level name	Number of units	Total population
1979	5	Sub-location	191	1,014,567
1989	5	Sub-location	212	1,444,296
1999	6	Enumeration area	2457	1,949,264
2009	5	Sub-location	279	2,679,478

### 2.4. Settlement extents

The beta version of the GHSL produced by the Joint Research Center of the European Commission was used as main temporally explicit predictor for our multi-temporal population mapping. The GHSL database uses the Global Land Survey collection of Landsat imagery produced by the Global Land Cover Facility (www.landcover.org) and the Landsat-8 data downloaded from the EarthExplorer website of the United States Geological Survey to depict settlement extents for four different epochs (1975, 1990, 2000, 2014) based on machine learning techniques (Pesaresi et al. 2013, 2016). Here we used the 300 m resolution aggregated dataset which represents the percentage of built-up area in each 300 × 300 m grid cell. In the GHSL database, settlement extents or built-up areas are defined as areas where the presence of built-up structures and buildings is observable from satellite data. Buildings include all constructions above ground intended for human or animal sheltering or for the production of economic goods. These constructions can be permanent or not, and therefore also include refugee camps or any other temporary settlement (Pesaresi et al. 2013).

In order to match the census years, a simple linear interpolation method was used to estimate the percentage of built-up area by 300 m grid cell for each census year (1979, 1989, 1999 and 2009), using the following equation:
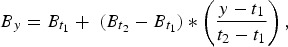
where 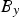 is the percentage of built-up area in target year 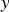, 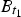 is the percentage of built-up area in the year of GHSL data preceding the target year (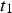) and 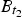 is the percentage of built-up area in the year of GHSL data following the target year (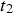). In the absence of information about potential irregularities in settlement growth, the linear interpolation method was chosen as it does not require additional information or assumption. Figure 1(b) and 1(c) shows the estimated percentage of built-up area for the four census years: 1979, 1989, 1999 and 2009.

### 2.5. Other geospatial datasets

Following methods described in Gaughan et al. (2016), we used a reduced set of covariates, limited to those that are time-invariant or temporally explicit. First, the global ESA CCI Land Cover database (© ESA Climate Change Initiative – Land Cover project 2014) was used to extract non-built-up land cover variables that correlate with the human population distribution. The ESA CCI database, which is derived from Medium Resolution Image Spectrometer imagery at a spatial resolution of 300 m, is an updated version of the GlobCover dataset that was previously used for mapping population distribution in Africa (Linard, Gilbert, and Tatem 2011; Linard et al. 2012). Non-built-up land cover classes were aggregated and re-coded to be consistent with those land cover classes used in previous AfriPop and WorldPop methodologies (Linard, Gilbert, and Tatem 2011; Linard et al. 2012; Stevens et al. 2015). The ESA CCI database provides land cover datasets for three different periods, centred around 2000, 2005 and 2010. While temporally explicit land cover data were used for 1999 and 2009, we had to assume the non-built-up classes to be time-invariant between 1979 and 1999 and therefore use the 2000 ESA CCI database for that period. While certainly not entirely correct, this is however a reasonable assumption given that we only consider nine aggregated non-built-up land cover classes: (1) cultivated lands, (2) woody/trees, (3) shrubs, (4) herbaceous, (5) sparse vegetation, (6) aquatic vegetation, (7) bare areas, (8) water bodies, (9) no data, cloud/shadow. Under these conditions, we assume that no major land cover change in non-built-up classes was observed in the study area over the 1979–1999 period.

Apart from the settlement layer and the non-built-up land cover classes described above, other geospatial datasets were assumed to be time-invariant in the study area and therefore included as covariates. Digital elevation data and its derived slope estimates were extracted from the Shuttle Radar Topography Mission HydroSheds data (Lehner, Verdin, and Fund 2006). In addition, we also included geospatial data that may correlate with human population presence such as networks of primary roads and waterways and protected areas, all of which are considered time-invariant. Different data sources were compared and the most comprehensive and temporally consistent datasets were selected, that is, permanent rivers from the National Geospatial-Intelligence Agency (NGA) Vector Map Level 0 (VMAP0) data (NGA 2005) and main roads from OpenStreetMap (OSM 2015). These main roads only include the Nairobi–Mombasa road that crosses the study area from west to east and the coastal road that links Mombasa with the Somalia border. Given their commercial importance, these roads were constructed several decades ago and were certainly already existing in the 1970s. Three recognized protected areas where human settlements are prohibited were digitized from Google Earth.

The predictors used in the present paper are summarized in Table 2. All data were converted to raster format and resampled by nearest neighbour to a square grid cell resolution of 3 arc seconds (approximately 100 m at the equator) in order to be included in the modelling framework described below.

**Table 2.** Summary of covariates used for population density estimation for each census year.

Type	Description	1979	1989	1999	2009
Settlements	Built-up percentage	GHSL 1979	GHSL 1989	GHSL 1999	GHSL 2009
Land cover	‘Class’ and ‘distance-to’ variables for each LC class, as defined in Stevens et al. (2015)	ESA CCI 2000	ESA CCI 2000	ESA CCI 2000	ESA CCI 2010
Roads	Distance to roads	OSM: main roads (OSM 2015)			
Rivers	Distance to permanent rivers	VMAP0 (NGA 2005)			
Elevation	Elevation and slope	HydroSHEDS (Lehner, Verdin, and Fund 2006)			

### 2.6. Population modelling approach

The RF modelling approach presented above was used to redistribute population counts within administrative units. Note that people were excluded from protected areas and from 200 m buffers around the coastline and main roads. Covariate importance was estimated using the percent increase in the mean squared error (MSE) evaluated with out-of-bag error when the covariate is randomly permuted (Breiman 2001; Liaw and Wiener 2002).

**Figure 2.**
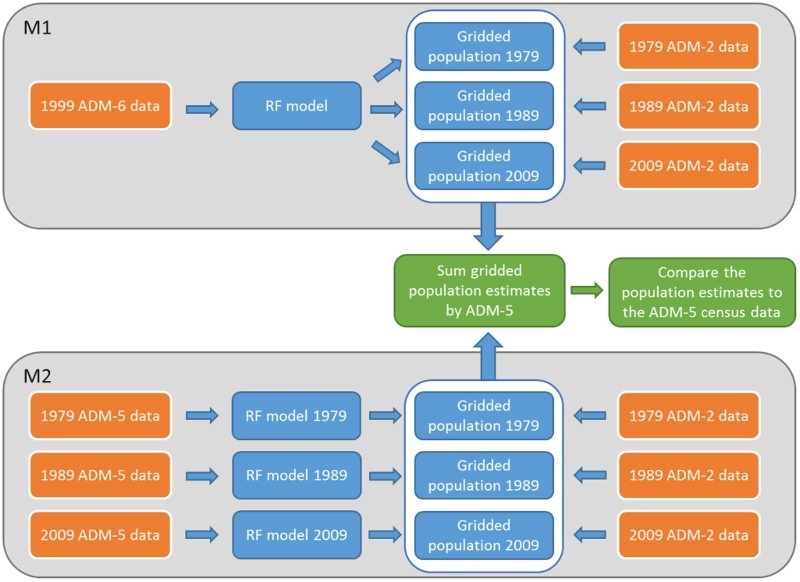
Flow diagram showing the processing steps used to compare the two models M1 and M2, including input census data in orange boxes, model outputs in blue boxes and validation steps in green boxes.

The finest level census data available are usually used for model estimation and prediction. However, previous work showed that when the spatial resolution of input census data was coarser than a certain threshold, using a regionally parameterized model provided more accurate predictions than country-specific models (Gaughan et al. 2014). Here, given that different levels of administrative units were available for the different years (Table 1), we tested the model predictive accuracy when using two different training datasets: (1) the most spatially detailed data (i.e. 1999 ADM-6 data) and (2) the most temporally relevant data for each year (i.e. ADM-5 data for 1979, 1989, 1999 and 2009). These two options are hereafter called Model 1 (M1) and Model 2 (M2) and summarized in Figure 2. In M1, a RF model is parameterized on finer-scale census data from a specific year and then applied to multiple years. There is an underlying assumption in M1 that the relationships between prediction density and covariates do not change over time. In contrast, in M2, each year is estimated independently from the others, meaning that changes in relationships between prediction density and covariates are taken into account.

For model comparison purposes, the dasymetric weighting layers obtained with the different models M1 and M2 were used to redistribute the coarser ADM-2 (district) populations of 1979, 1989 and 2009, as illustrated in Figure 2. ADM-2 data were obtained by aggregating the ADM-5 data using district identifiers. We then compared the output maps to the ADM-5 census data for each year by summing gridded population estimates within each administrative unit. Accuracy assessment statistics were calculated to compare the performance of the models for the three census years with finest ADM-5 census data (1979, 1989 and 2009), including root mean squared error (RMSE) and the mean absolute error (MAE). These statistics were used to identify the best model estimation approach (M1 or M2) that was then used to predict population distributions for each census year.

## 3. Results

**Figure 3.**
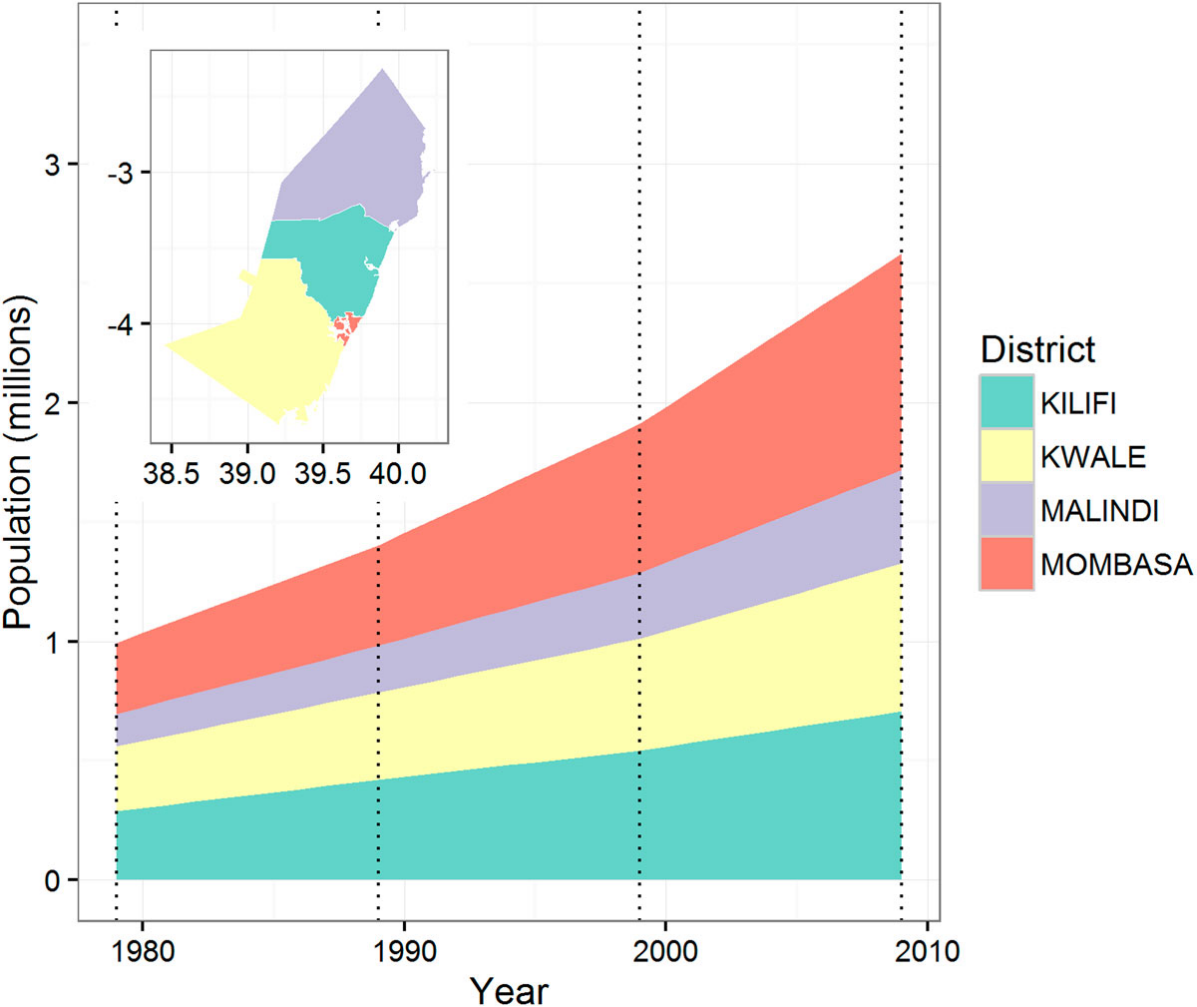
Population growth for coastal districts 1979–2009, based on population counts from the 1979, 1989, 1999 and 2009 censuses. Dotted vertical lines show the census years.

Figure 3 shows the population growth in the Kenyan coastal districts between 1979 and 2009. The total population multiplied by more than 2.5 during the period with a higher average annual growth rate in the urban district Mombasa (3.71%) compared to the rural districts Kilifi (2.98%), Kwale (2.76%) and Malindi (3.52%).

**Figure 4.**
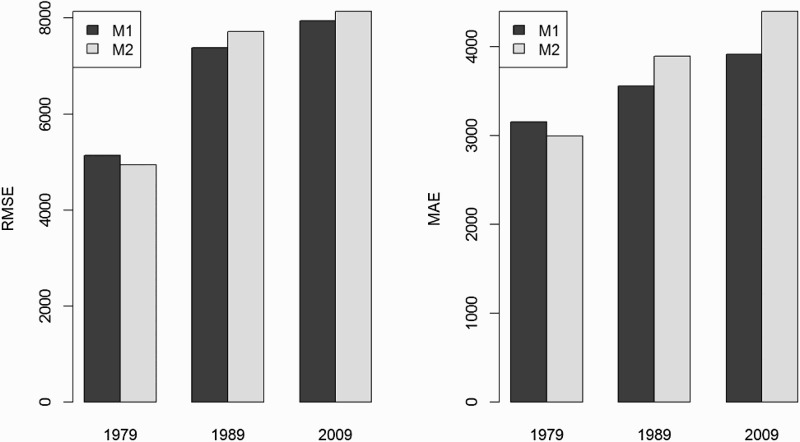
Comparison of accuracy statistics (RMSE and MAE) of the two different modelling approaches M1 and M2.

Figure 4 compares the performance of the two different models M1 and M2 for predicting population distributions in 1979, 1989 and 2009. Results show that M1 was more accurate for the years 1989 and 2009, while M2 was more accurate for 1979, the most temporally distant year from training data. Results are consistent between statistics used (RMSE and MAE). The best model estimation approach therefore varies between years. M1 takes advantage of the more spatially detailed ADM-6 census data available for 1999 but assume that relationships between environmental variables and population distribution are stable over time. M1 should therefore be favoured when modelling over limited time windows. On the contrary, M2 takes advantage of having different census dates and develop specific models for each census year. M2 should be favoured for large time windows, provided that the spatial detail of census data is sufficient for training a model.

**Figure 5.**
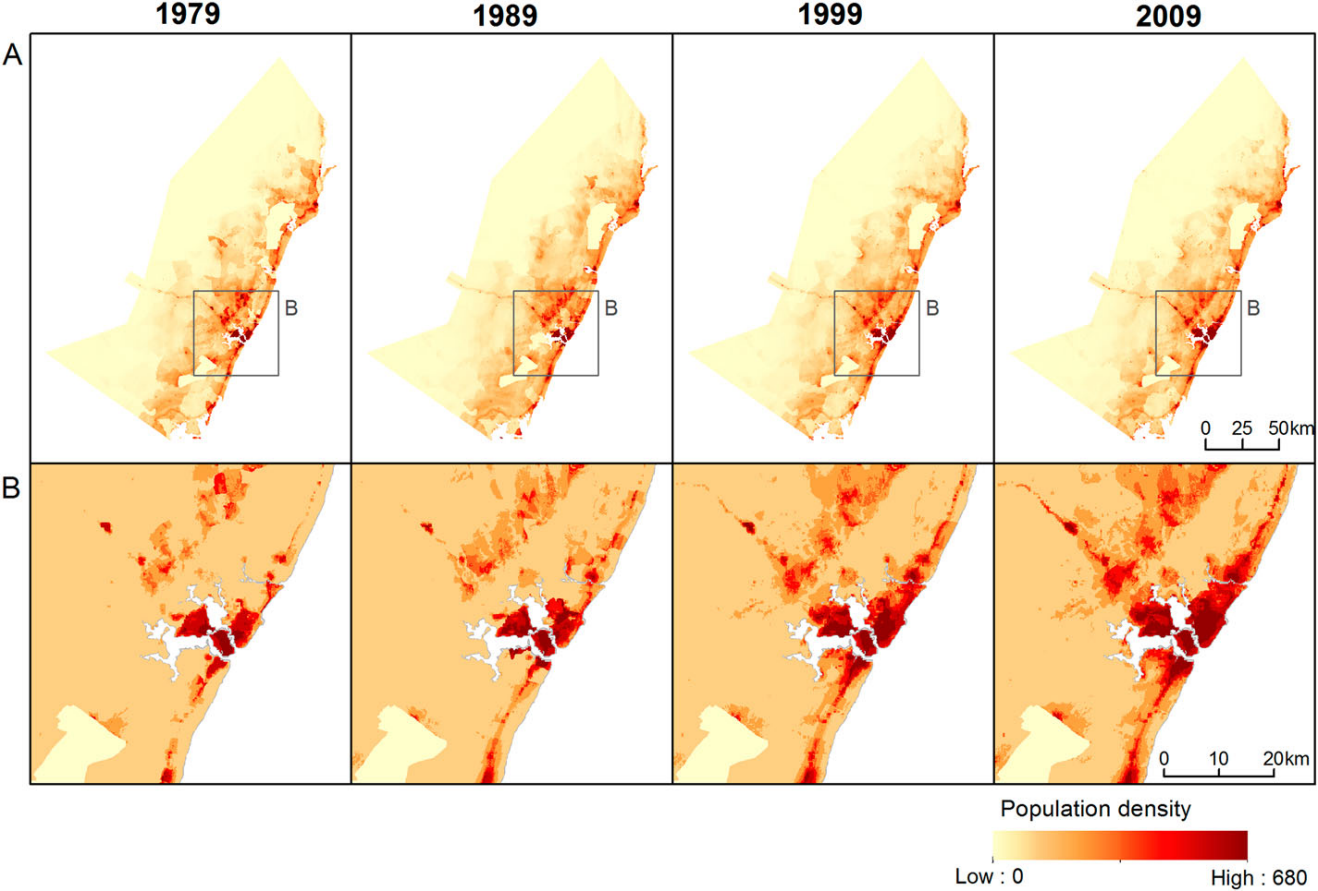
Predicted population density in the coastal districts of Kenya for the four census years (1979, 1989, 1999 and 2009) using M1 for (a) the whole study area and (b) close-ups around Mombasa. Grid cell resolution is 3 arc seconds, or ∼100 m at the equator, and grid cell values represent people per hectare.

Here, for consistency purposes, final population distribution datasets were produced using M1, that is, using the most spatially detailed data (1999 ADM-6 data) for training the model, even if the best model estimation approach varies between years. Population distribution datasets for the census years are shown in Figure 5.

**Figure 6.**
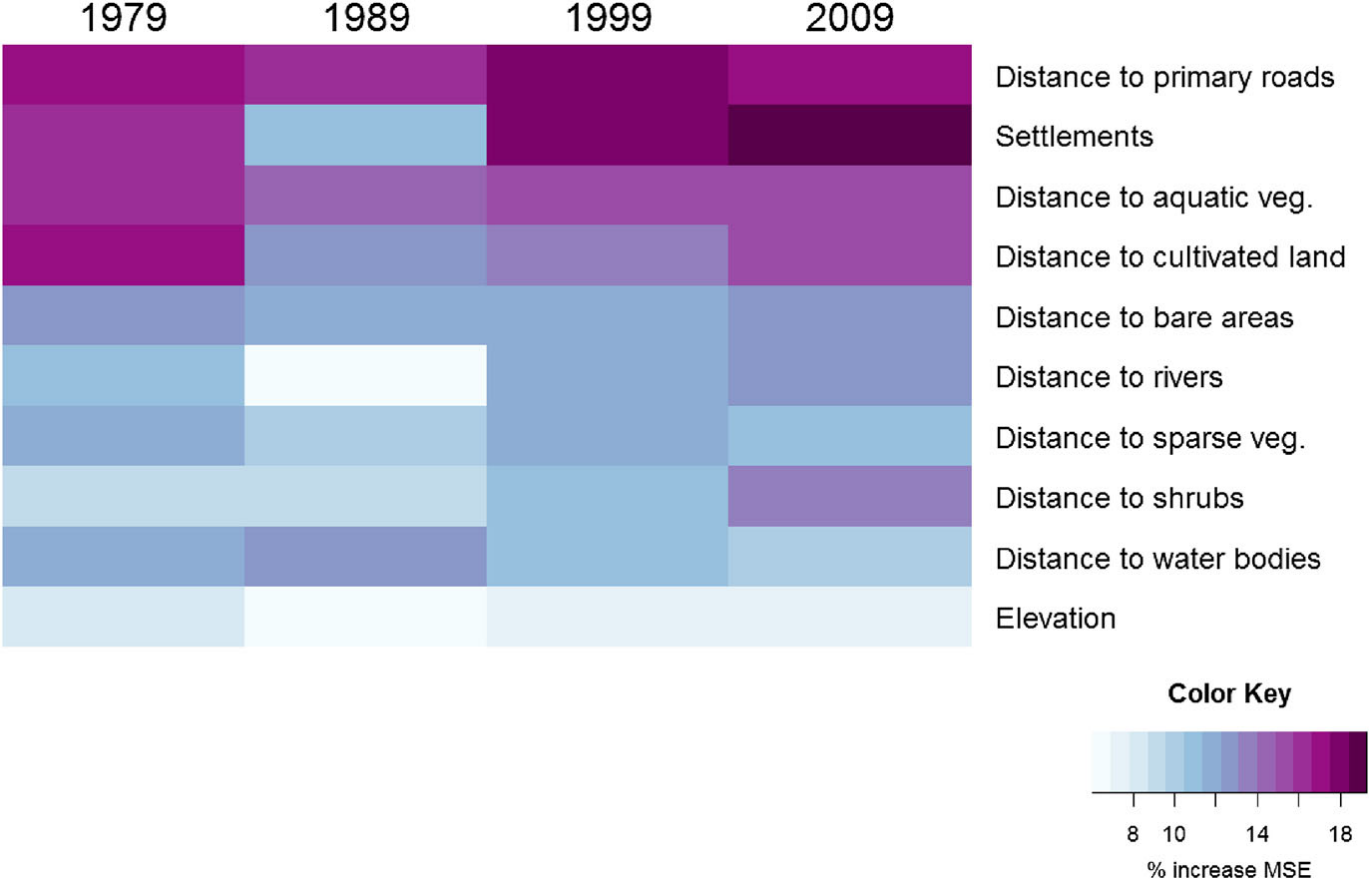
Percent increase in the MSE when the covariate is randomly permuted in the different year-specific M2 models.

Having different RF models in the M2 approach allows the comparison of covariate importance between years and therefore the evaluation of the stability of statistical relationships through time. In almost all models, settlements were among the most important predictors (Figure 6). A noticeable exception is the 1989 model, where settlements have been supplanted by several other covariates such as the distance to roads and distance to different land cover classes (Figure 6). The increase in the MSE ranges between 11% and 20% when the settlement variable is removed from M2 models. Other consistently important covariates include the distance to primary roads, distance to natural land cover classes and distance to cultivated land. More generally, Figure 6 shows that, apart from a few exceptions (e.g. settlements in 1989), the importance of variables is relatively stable between M2 models, which suggests that relationships between environmental variables and population distribution are quite stable over time.

## 4. Discussion

When modelling population distribution changes, three different temporally varying elements have to be considered: (a) the changing population counts by administrative unit, (b) the changing geographical covariates and (c) the changing relationships between population distribution and geographical covariates. When projecting backward in time, (a) and (b) are sometimes available, though not always at the same spatial and temporal resolutions, which complicates the evaluation of (c). The present case study represents a typical situation of varying spatial resolutions of available census data between years. Model comparisons showed that trade-offs have to be found between spatial and temporal resolutions when selecting the best modelling approach. In the present case study, we found that the best approach varied through time. Here, we favoured the model parameterization using finer-scale census data from a specific year and the application of this single model to multiple years, with the underlying assumption that the relationships between prediction density and geographical covariates do not change over time. However, a different approach was preferred for China (Gaughan et al. 2016). In this specific region of the World and for a specific time period (1990–2010), each year was estimated independently from the others, meaning that changes in relationships between prediction density and covariates were taken into account (Gaughan et al. 2016). In the China case study, however, the number of administrative units available was comparable for each census. In the present case study, given the large differences in the number of census units available for training the RF model (i.e. 2457 in the ADM-6 data used in M1 and between 191 and 279 in the ADM-5 data used in M2), it seemed more adequate to take advantage of the very detailed census data than using temporally explicit census data for training the RF model. There is no consensus on the best approach, as it depends on the spatial and temporal resolution of input population data, but also on local characteristics. While in China, the fast urban development has been associated with large national policy reforms, in Kenya, people are more inclined to settle where they like, without influence from regulations.

The present paper reveals the methodological challenges that need to be tackled when trying to model population distribution changes in data-scarce countries. Data gaps need to be filled using strategies that may vary according to the local context and the objective of the study. While we expect the relationships between population distribution and geographical covariates to vary in space and time, we need to assume these relationships to be constant as soon as we start making predictions in spatial and temporal windows for which good census data are not available. Our results showed that the importance of covariates did not radically change through time in our study area, which suggest relatively stable relationships between population distribution and environmental covariates through time. In the present case study, assuming these relationships to be constant allowed us taking advantage of higher resolution data and resulted in more accurate outputs. Similarly, if we want to make population distribution predictions for the future that take predicted urban land cover changes into account (Linard, Tatem, and Gilbert 2013), we do not have any other choice than assuming constant statistical relationships between population distribution and geographical covariates.

The consistency between datasets is another factor to take into account. In many applications, for example when estimating changes in epidemiological risks, temporally comparable datasets are essential. This requires the use of a limited number of covariates that are time-invariant or temporally explicit (Gaughan et al. 2016). While lights at night are useful datasets for producing multi-temporal population density maps, such data are limited to the last 2.5 decades. Built-up areas are also key predictors of population distribution and the increasing availability of earth observation data allows to go back further in time. However, the lack of spatially detailed time-series of built-up areas has so far prevented the production of population distribution time-series. Here we made use of the new high-resolution GHSL dataset which depicts settlement extents globally and made settlement extent predictions for every year by assuming linear settlement changes between 1979 and 2009. This new dataset, combined with different census data, allowed the production of gridded population distribution dataset for a period of 30 years in the Kenyan coastal districts and is extremely promising for future developments of multi-temporal population datasets at larger spatial scales. However, to estimate built-up density at specific dates (i.e. census years), assumptions have to be made on the evolution of built-up density to fill temporal data gaps. Here, we assumed linear changes between years as no additional temporal information on built-up evolution was available. More sophisticated interpolation methods could be used if allowed by data availability, but could also introduce other bias and assumptions. In any case, further analyses would be needed to better evaluate the accuracy of GHSL for different time periods and accuracy losses due to the interpolation method used. Future work will focus on more detailed evaluations of the usefulness of new settlement layers such as GHSL or the Global Urban Footprint (Esch et al. 2013, 2014) for population mapping purposes. Other currently ongoing research areas may help to improve future population distribution models in data-scarce circumstances. First, mobile phone data may be used to improve the temporal resolution of population datasets and may be particularly useful for modelling short-term population changes (Deville et al. 2014). Secondly, bottom-up population mapping approaches that use a combination of satellite-derived features and household surveys for estimating population may be particularly useful where census data are unreliable. Finally, new tools like Google Earth Engine may significantly improve the potential of rapid settlement and population mapping by improving capabilities of analysing and processing large amounts of remotely sensed images (Patel et al. 2015).

The construction of multi-temporal gridded population datasets has been described here for a relatively small study area and highlighted some of the methodological challenges and opportunities for future larger-scale development of time-series in population distribution modelling.
